# Evolutionary history of the *NAM-B1* gene in wild and domesticated tetraploid wheat

**DOI:** 10.1186/s12863-017-0566-7

**Published:** 2017-12-20

**Authors:** Maria Lundström, Matti W. Leino, Jenny Hagenblad

**Affiliations:** 1 Linköping University, IFM Biology, SE-581 83 Linköping, Sweden; 2 Nordiska museet, Swedish Museum of Cultural History, Box 27820, SE-115 93 Stockholm, Sweden; 3 The Archaeological Research Laboratory, Department of Archaeology and Classical Studies, Stockholm University, SE-106 91 Stockholm, Sweden

**Keywords:** Selective sweep, Grain protein content (GPC), Emmer, Durum, Domestication gene

## Abstract

**Background:**

The *NAM-B1* gene in wheat has for almost three decades been extensively studied and utilized in breeding programs because of its significant impact on grain protein and mineral content and pleiotropic effects on senescence rate and grain size. First detected in wild emmer wheat, the wild-type allele of the gene has been introgressed into durum and bread wheat. Later studies have, however, also found the presence of the wild-type allele in some domesticated subspecies. In this study we trace the evolutionary history of the *NAM-B1* in tetraploid wheat species and evaluate it as a putative domestication gene.

**Results:**

Genotyping of wild and landrace tetraploid accessions showed presence of only null alleles in durum. Domesticated emmer wheats contained both null alleles and the wild-type allele while wild emmers, with one exception, only carried the wild-type allele. One of the null alleles consists of a deletion that covers several 100 kb. The other null-allele, a one-basepair frame-shift insertion, likely arose among wild emmer. This allele was the target of a selective sweep, extending over several 100 kb.

**Conclusions:**

The *NAM-B1* gene fulfils some criteria for being a domestication gene by encoding a trait of domestication relevance (seed size) and is here shown to have been under positive selection. The presence of both wild-type and null alleles in domesticated emmer does, however, suggest the gene to be a diversification gene in this species. Further studies of genotype-environment interactions are needed to find out under what conditions selection on different *NAM-B1* alleles have been beneficial.

**Electronic supplementary material:**

The online version of this article (10.1186/s12863-017-0566-7) contains supplementary material, which is available to authorized users.

## Background

The domestication of tetraploid wheats began more than 10,000 years ago [1, 2]. The process originated in the Fertile Crescent, where stands of wild tetraploid emmer (*Triticum turgidum* subsp. *dicoccoides*) can still be found (reviewed in [3]). The circumstances surrounding the domestication of the tetraploid wheats, resulting in domesticated emmer (*Triticum turgidum* subsp. *dicoccum*) and durum wheat (*Triticum turgidum* subsp. *durum*), have long been debated. Some studies suggest a monophyletic origin of domesticated wheat [4, 5], possibly with gene flow between wild and domesticated populations [5], while others propose a polyphyletic origin [6, 7]. More recently it has been suggested that domesticated tetraploid wheats arose from a mixed set of wild emmer populations [8]. The hexaploid wheats, including bread wheat *Triticum aestivum* subsp. *aestivum*, arose as a result of hybridization between free-threshing *Triticum turgidum* and the wild diploid *Aegilops tauschii* [9, 10]. There also appears to have been introgression of genetic material from wild tetraploid populations into hexaploid wheat, which could have happened either post-domestication or via the tetraploid progenitor [10–12].

Domestication of wheat affected several traits, most notably leading to the evolution of a non-brittle rachis (under control of two major genes: *Br-A2* and *Br-A3* [13]). Other traits in the domestication syndrome are free-threshing (influenced by the genes *Q* and *Tg*) [14, 15] and increased seed size [16]. The latter is influenced by the gene *Gpc-B1*, also known as *NAM-B1*, which has consequently been suggested to be a domestication gene [17]. In order to be considered a domestication gene three criteria should be fulfilled: it should underlie a trait associated with domestication, it should have experienced positive selection, and it should be at near or complete fixation for the causative allele behind the trait [18].

The function of *NAM-B1* is well characterized as a NAC transcription factor [19]. Three alleles have been described of which only the wild-type (henceforth WT) is functional. The other two, a one-basepair insertion frameshift mutation (henceforth +1 bp) and a deletion (henceforth del) of unknown size, are null alleles. The WT allele accelerates senescence and facilitates remobilization of nutrients from flag leaf into maturing grain [20–24]. This results in grain that has higher concentrations of protein and micronutrients [22, 25–28]. In contrast, the null alleles delay senescence, which can prolong the grain-filling period and thereby increase seed size [26]. Whereas the WT allele has been found to increase protein and mineral content throughout environments, the effect on seed size is more dependent on genetic background and environment [26]. Distelfeld, et al. [22] suggested that the seed size enhancing effects of the null alleles are more pronounced in cooler, wetter climates that allows a longer grain-filling period.

In a study by Uauy, et al. [19] the WT allele of *NAM-B1* was found to be present in all investigated wild emmers and in most of the domesticated emmers, while it was absent in the bread or durum wheats. The +1 bp allele was found in all durum wheats and a few of the domesticated emmers and bread wheats, while most bread wheats appeared to carry the deletion. Based on this screening the authors concluded that the WT allele was lost during domestication of the progenitors of bread wheat and durum [19]. Asplund, et al. [29], however, could show that the WT allele was present in four historical specimens of hexaploid landrace wheats. This suggested that the absence of the WT allele in modern wheat varieties was at least partly the result of more recent selection. The presence of the WT allele was later confirmed in extant hexaploid wheats where it was prominent among Fennoscandian landrace spring wheats [30]. Based on their global distribution, the null alleles could, however, arguably be considered to be near fixation in bread wheat.

Although many genes in various crops, including *NAM-B1* [17], have been proposed to be domestication genes, only a few have been shown to have been targeted by selection [31]. When exposed to selection not only the favored genetic variant increases in frequency, but also other, neutral, polymorphisms linked to it. The result is a genetic region with reduced genetic diversity [32, 33], a so-called selective sweep. It has been suggested that during domestication much, if not most, selection will act on already existing genetic variants that become beneficial when artificial selection is applied [34]. The resulting soft sweeps, are expected to leave more subtle traces in the genome than selection acting on *de novo* mutations [18, 34] and classic tests for selective sweeps will have limited power to detect soft sweeps. Evidence of selection is, however, needed to be able to call a gene a domestication gene [18]. In order to further investigate *NAM-B1*’s previously suggested status as a domestication gene [17, 35–37], we therefore set out to determine whether we could detect traces of selection in the *NAM-B1* region of tetraploid wheat with the +1 bp allele, and whether the strength of selection acting on the gene could be estimated. In addition we sought to determine the size of the deletion allele.

## Methods

### Plant materials

Two different sets of plant materials were used (Additional file 1). The first set was used to determine the size of the deletion. It consisted of six hexaploid wheats with known *NAM-B1* genotypes; ‘Anza’ (del), ‘Glenlea’ (del), ‘Little club’ (del), ‘Chinese spring’ (+1 bp), ‘Aurore’ (+1 bp) and PI 350731 (WT), with the nullisomic line N6BT6D used as a control to exclude amplification of homologous regions on chromosomes other than 6B.

The second set was used to investigate the presence of a selective sweep in the *NAM-B1* region. In a first screen, 94 tetraploid landrace wheats were genotyped for the *NAM-B1* gene. The materials primarily originated from the Mediterranean and Near East, and comprised of 18 wild emmers, 61 domesticated emmers and 15 durum wheats. From these, 40 wheats were chosen for the second set of plant materials, which also included six hexaploid wheats used in a previous study [30] and five outgroup accessions of *Aegilops speltoides*, *Aegilops tauschii* and *Triticum monococcum*. The tetraploid accessions were chosen so that, whenever possible, different genotypes were represented among the different subspecies: ten domesticated emmers with WT and ten with +1 bp allele, ten wild emmers with WT and one with +1 bp, and nine durum wheats all of which carried the +1 bp allele.

DNA was extracted from leaf tissue using either DNeasy Plant Mini Kit (Qiagen) or E-Z 96 Plant DNA kit (Omega bio-tek).

### PCR amplification

**Fig. 1:**
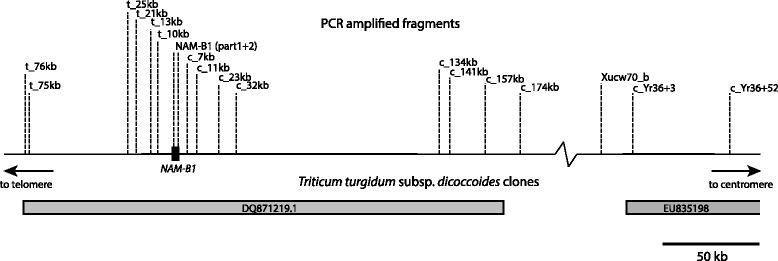
PCR-fragment positions relative to *NAM-B1*

Primers (Additional file 2) targeting regions within and around *NAM-B1* (Fig. 1) were used to determine whether each particular region was deleted or not in the first set of plant materials. Lack of amplification in ‘Anza’, ‘Glenlea’ and ‘Little club’ but with successful amplification in ‘Chinese spring’, ‘Aurore’ and PI 350731 was interpreted as that region being deleted.

The 94 tetraploid wheats were genotyped for *NAM-B1* according to Asplund, et al. [29]. The resulting PCR fragments were run on an ABI 3130xl Genetic Analyzer with MegaBACE ET400-R Size Standard. Accessions showing no amplification after multiple PCR attempts, but where amplification outside the deleted region verified DNA quality, were considered to carry the deletion.

The second set of plant materials was used to look for signs of a selective sweep. Nine fragments in *NAM-B1* and the surrounding genomic region on chromosome 6B were amplified using PCR (Additional file 2). Four reference genes; *MdhA*, *Mp7A*, *Gsp1B*, and *11B* [4], believed to be unlinked to *NAM-B1,* were included for comparison. PCR specificity to chromosome 6B was verified by failure to amplify DNA from the nullisomic line N6BT6D.

For PCR amplification in both sets each reaction contained 0.05 U/μl of DreamTaq DNA polymerase (Thermo Scientific), 1 x DreamTaq Buffer (Thermo Scientific), 0.2 mM of each dNTP (Thermo Scientific), 0.1 μM each of forward and reverse primer, respectively, and 1 μl of DNA template. PCR conditions were as given in Additional file 2. Unincorporated nucleotides and primers were removed from the PCR products using 0.014 U/μl Exonuclease I (Thermo Scientific) and 0.0071 U/μl FastAP Thermosensitive Alkaline Phosphatase (Thermo Scientific) incubated at 37 °C for 30 min followed by 5 min at 95 °C. Sequencing was performed by Eurofins MWG Operon, Germany, and Macrogen Europe, The Netherlands.

### DNA sequence analysis

Geneious (6.0.5) was used to edit and align the DNA sequences and for constructing neighbor-joining trees (Tamura-Nei). DnaSP (ver 5.10.01) [38] was used to test for selection by calculating Tajima’s D [39] and Fu and Li’s D and F [40] statistics. Where outgroup data was not available Fu and Li’s D^*^ and F^*^ statistics were calculated instead. The strength of selection acting on *NAM-B1* was estimated according to Olsen, et al. [33].

## Results

### The deletion allele

Of the three known alleles at *NAM-B1* one is a deletion. By PCR-amplifying targets in the region surrounding the gene (Fig. 1) in six hexaploid wheats with known *NAM-B1* genotypes we found that the deletion covered a fragment located 25 kb away from *NAM-B1* on the telomeric side of the gene (henceforth t_25kb) but not the t_75kb fragment. On the centromeric side of the gene the deletion covered a fragment located 174 kb away from *NAM-B1* (henceforth c_174kb). We did, however, find amplification in one accession carrying the deletion (‘Anza’) at Xucw70_b located between BAC DQ871219.1, that contains *NAM-B1*, and the adjacent BAC EU835198 located less than 0.3 cM away from *NAM-B1*. The two other accessions with the deletion (‘Glenlea’ and ‘Little club’) did not show any amplification until c_Yr36 + 3, located within EU835198 but further away on the centromeric side, suggesting that the region contains multiple deletions. In conclusion, the deletion was found to cover a region of more than 200 kb, stretching from approximately 25 kb on the telomeric side and past a fragment located 174 kb into the centromeric side of *NAM-B1*.

### Allele distribution in tetraploid wheats

**Fig. 2:**
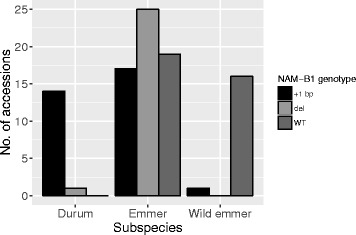
Distribution of *NAM-B1* genotypes in tetraploid wheat subspecies

We screened 94 accessions of wild and landrace tetraploid wheats for *NAM-B1* alleles. All 18 wild emmer accessions carried the WT allele except PI 355459 that carried the +1 bp allele and PI 233288 which was found to be heterozygous and was hence excluded from further study. In contrast, all the 15 durum wheats had the +1 bp allele except TRI 14552, which carried the deletion. Among the domesticated emmer accessions 25 out of the 61 genotyped carried the deletion, 17 had the +1 bp allele and 19 carried the WT allele (Fig. 2). Neither of the *NAM-B1* alleles appeared to be limited to specific geographic areas. The WT allele was frequently found in accessions from the Middle East, reflecting that this is the most common allele among the wild emmers that are native to this region.

### Phylogenetic and diversity analyses in the region surrounding NAM-B1

**Fig. 3:**
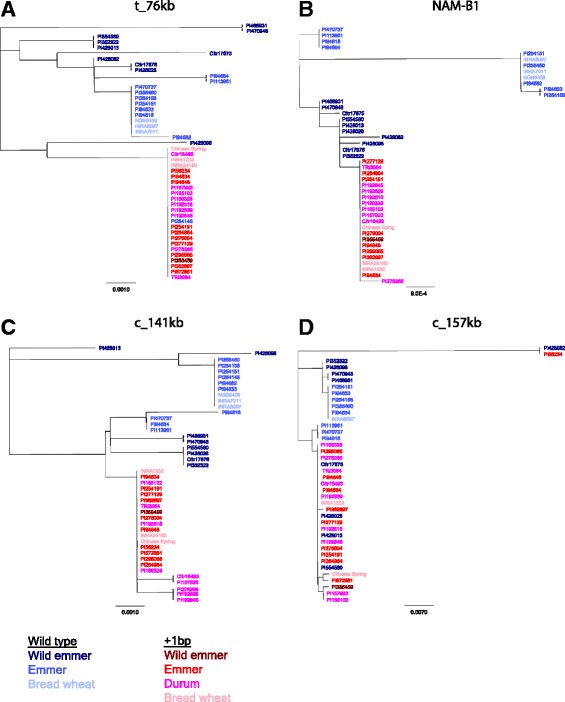
Neighbor-joining trees of wheat sequences in fragments at different distances from *NAM-B1*. **a** Starting on the telomeric side of the *NAM-B1* gene with fragment t_76kb, **b** followed by *NAM-B1*, **c** and then into the centromeric side in c_141kb **d** only to show no clustering according to *NAM-B1* genotype in fragment c_157kb

Fragments across the *NAM-B1* region were sequenced in the second sample set to investigate the presence of a selective sweep. Neighbor-joining trees showed that the sequences formed two clusters; one consisting of WT accessions and another with accessions carrying the +1 bp allele both within the gene as well as outside of it (Fig. 3a-c) from t_76kb to c_141kb. The WT accessions were fairly polymorphic and in *NAM-B1* the less variable +1 bp accessions formed a subclade within a group of WT wild emmers. In c_7kb (Additional file 3) and t_76kb (Fig. 3a) the occasional WT accession clustered among the +1 bp accessions. In c_157kb (Fig. 3d) accessions no longer clustered according to allele type, in concordance with the four reference genes.

**Fig. 4:**
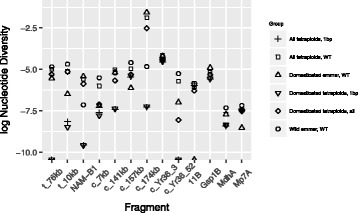
Nucleotide diversity. Nucleotide diversity (log transformed) in the fragments surrounding *NAM-B1* and the four reference genes

The nucleotide diversity of the sequences from sample set two was examined with sequences being grouped by *NAM-B1* genotype as well as by domestication status and subspecies (Fig. 4, Additional file 4 a–b). In all but three fragments (*NAM-B1,* one linked and one reference fragment) nucleotide diversity (π) was higher in wild emmer than in the domesticated wheats combined (three fragments for θ; *NAM-*B1, one linked and one reference). Inclusion of the +1 bp wild emmer had little effect on the diversity measures. Nucleotide diversity (π) was higher in WT accessions than in +1 bp accessions in all fragments. When comparing only domesticated wheats the WT nucleotide diversity (π) was higher than +1 bp diversity in all but three fragments; two reference fragments and one linked to *NAM-B1* (three fragments for θ; two linked and one reference).

### Tests for selection

**Fig. 5:**
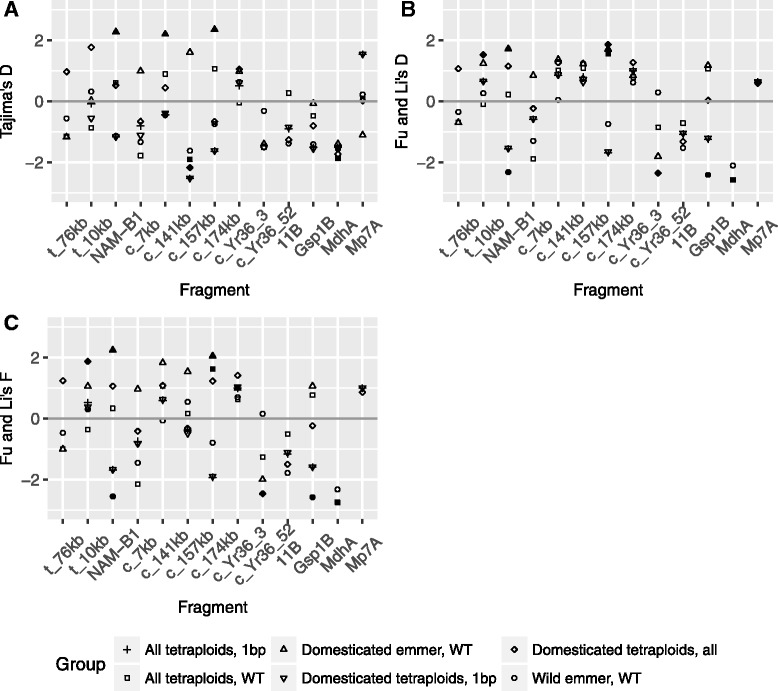
Neutrality test values. The plot contains the D and F values resulting from the neutrality tests in the different fragments around *NAM-B1* and the reference genes. Filled symbols indicate significance of at least *p* < 0.05. **a** Tajima’s D. **b** Fu and Li’s D or D^*^. **c** Fu and Li’s F or F^*^

The sequences from sample set two were also analyzed to see if any of the fragments showed significantly higher frequencies of rare alleles than expected under neutral theory, indicative of a selective sweep. Such an excess of rare alleles would reveal itself as negative test statistics when tested with Tajima’s D or Fu and Li’s D and F. For the four reference fragments both wild emmers and domesticated tetraploid wheats primarily produced negative test statistics (Fig. 5a-c, Additional file 5a–c). Wild emmers likewise tended to produce negative test statistics also in the fragments surrounding the *NAM-B1* at least on the telomeric side and when tested with Tajima’s D. For most fragments test statistics continued to be negative after the inclusion of the +1 bp wild emmer. Domesticated wheats, in contrast, had in general positive test statistics throughout the *NAM-B1* region.

When separated according to genotype, both domesticated wheats with WT and those with +1 bp had negative Tajima’s D values (Fig. 5a; Additional file 5a) for three out of the four reference fragments. Fu and Li’s test statistics were positive for the WT accessions in the reference gene from which results were obtained, and negative in two of the fragments for the +1 bp accessions (Fig. 5b-c, Additional file 5b–c). For the fragments in the *NAM-B1* region Tajima’s D values were in general negative for the +1 bp accessions and positive for the WT accessions. In *NAM-B1* itself this was the case for all three test statistics, with significant positive values for WT accessions, but the pattern was less clear for Fu and Li’s D in the fragments surrounding *NAM-B1*. In conclusion, selection test statistics tended to be negative in all species and allele groups with the exception of domesticated WT accessions for fragments in the *NAM-B1* region.

In wheat, the markers *Xucw70* and *Xucw73*, flanking the *NAM-B1* region, are located 0.9 cM apart, corresponding to a 100 kb segment in rice [41]. Using these values as an approximate estimate of the recombination frequency in the region and 141 kb as an estimate of the width of the selective sweep we followed Olsen, et al. [33] and estimated the strength of selection acting on *NAM-B1* to 0.13.

## Discussion

### Genetic effects of tetraploid wheat domestication

Domestication is expected to cause a genome-wide bottleneck with a resultant loss of genetic diversity [31, 42]. Consequently, wild emmer is expected to contain higher levels of genetic diversity than domesticated wheats [43–45]. Overall, we found higher levels of diversity in wild emmer than in domesticated wheats, both for the reference genes and to a higher extent in the *NAM-B1* region, supporting the presence of a domestication bottleneck. The levels of genetic diversity detected in the reference fragments, although moderate, were similar or higher to those previously reported from the same genes also in wild emmer [4]. We thus conclude that our sample of wild emmer well describes the diversity currently available in genebank holdings.

When comparing the two domesticated tetraploid wheat species, emmer and durum, the former tended to have higher diversity. This is in agreement with previous studies where low nucleotide diversity have been reported in domesticated wheats, in particular durum wheat [4, 46]. In contrast, in a study of Mediterranean wheats, Oliveira, et al. [12] found higher SNP diversity in the order: durum > domesticated emmer > wild emmer wheat, but noted that this may have been the consequence of ascertainment bias as the markers used had been developed from a panel of durum and rivet wheat.

For some of the fragments in this study, our unascertained sequences from domesticated emmer showed higher diversity than wild emmer, corroborating the results of Oliveira, et al. [12]. A reticulated origin of domesticated emmer wheats [8] or gene flow between wild and domesticated species [5, 11] could have contributed to increasing the nucleotide diversity in parts of the genome of domesticated wheat. It has been argued, however, that the effect of introgression from wild wheats may have been limited, in particular as it would have posed a risk to losing recessive domestication traits [8, 47].

Many tests for selection, such as the ones used here, are also sensitive to population expansion and subdivision. The negative (albeit rarely significant) test statistics detected for most of the four reference fragments suggest population expansion, both in wild and domesticated tetraploid wheats. Population expansion following the initial domestication bottleneck is expected in the domesticated wheats, as a consequence of spread of agriculture. The evolutionary explanation for the negative test statistics for wild emmer is less clear, but the results corroborate those of previous studies [4, 12].

### Null alleles at *NAM-B1*

There are two known null alleles at the *NAM-B1* locus, one of which is a deletion (del) [19]. We report here, for the first time, the presence of the del allele among durum wheats, which suggests that both null alleles occur in both durum and emmer landrace wheat. The rarity of the del allele among durum wheats (a single accession – TRI 14552) could be the result of genetic drift during a bottleneck during the formation of the species, but neither our reference genes nor the results of other studies have consistently suggested a strong bottleneck during durum formation [12, 48]. Further genetic analysis of the del carrying accession would be needed to rule out recent gene flow from domesticated emmer.

We have found that the deletion allele stretches over a region of at least 200 kb. Our data also suggest that more deletions occur in the same genetic region. A large proportion of the region surrounding *NAM-B1* consists of repetitive sequence, facilitating repeated loss of DNA [49]. This plasticity of the plant genomes, resulting from both repetitive DNA introducing variation and motifs involved in illegitimate recombination together with the functional overlap among genes in polyploids, has been suggested to be of major importance for crop evolution [17, 50–52]. In the case of *NAM-B1* it has given rise to one of the two null alleles.

Accessions with the +1 bp allele form a subclade to accessions with the WT allele (Fig. 3b). The subclade, however, falls under the wild emmers rather than WT accessions of the domesticated subspecies. This strongly suggests that the +1 bp mutation first arose in wild emmer rather than in the domesticated forms. This is further supported by the presence of the +1 bp allele in a wild emmer accession. This accession, PI 355459, has the spikelet morphology and smooth breakage scars consistent with it being a true wild emmer, although we cannot rule out that the presence of the +1 bp allele is the result of gene flow from domesticated tetraploids back into wild emmers.

### A selective sweep at NAM-B1

Genetic diversity at linked sites is expected to share a common genetic history, with alleles at different loci being non-randomly associated, in so-called linkage disequilibrium [53]. Recombination will act to break up such associations and gene trees at unlinked or loosely linked parts of the genome are typically independent of each other. Selective sweeps, such as those expected following selection on traits beneficial to domesticated plants, will act to increase the extent of linkage disequilibrium and the amount of linkage disequilibrium will depend both on the strength of the sweep and the time since the sweep occurred [54].

We found that accessions cluster according to *NAM-B1* genotype up to at least 141 kb on the centromere side and beyond 76 kb on the telomere side of *NAM-B1*, indicative of a large sweep. A sweep affecting a region of similar size (250 kb) has been reported to surround the *Waxy* (*Wx)* gene, causing the absence of amylose in glutinous rice [33], a primarily self-pollinating species like wheat. In contrast, the sweep located just upstream of *teosinte branched 1* (*tb1)* in maize, a cross-pollinating species, is much smaller at 65.6 kb [55–57]. In maize the sweep appears to have been targeting the regulatory region where domesticated maize carries a transposon insertion that alters the expression levels of *tb1* [57, 58]. This affects several downstream targets of *tb1* [59] resulting in, among other things, altered branching pattern. In the case of *Wx* the strength of selection was estimated to be on the order of 4.24–4.59 [33] while the inferred selection coefficient in maize was much weaker at no more than 0.08 [55, 56]. Our estimate of the strength of selection suggests that the +1 bp allele has been more strongly selected than *tb1* but less strongly selected than *Wx*.

In addition to increasing the extent of linkage disequilibrium, selective sweeps reduce the genetic diversity in the targeted region and its surrounding region. Consistent with this we found that the accessions of domesticated wheats carrying the +1 bp allele had lower levels of nucleotide diversity both in *NAM-B1* and in surrounding fragments than both wild emmers and domesticated tetraploid wheats carrying the WT allele (Fig. 4a). For the reference genes the diversity differences between the two allele-groups were typically smaller.

In spite of the high level of linkage disequilibrium and the low genetic diversity indicative of a selective sweep at *NAM-B1*, neutrality tests mostly failed to detect the sweep. This was in many cases due to the lack of genetic diversity, itself an expected outcome of a selective sweep (e.g. [60]), among the +1 bp accessions. Where genetic diversity was present among the +1 bp accessions neutrality tests often produced the negative test statistics indicative of a selective sweep (Fig. 5; Additional file 5) although the tests were rarely significant. The lack of significance is surprising, given not only the expected selective history of the gene. The population expansion suggested for domesticated tetraploid wheats by the reference fragments and, for emmer, by previous studies [12] should have pushed also the fragments in the *NAM-B1* region towards more negative test statistics.

### Evolutionary history of the NAM-B1 region

It has been suggested that three criteria should be met in order for a gene to be called a domestication gene [18]. The first criterion is that its function should be characterized and it should code for a trait of interest during domestication. Wheat carrying null alleles of *NAM-B1* has been shown to have slower senescence rate, resulting in an increased grain weight [21, 23, 26] and based on this we consider *NAM-B1* to fulfill the first criterion. Secondly there should be evidence of positive selection at the locus [18]. From the location of the +1 bp accessions in the gene tree for *NAM-B1* (Fig. 3b) we can deduce that the +1 bp allele likely arose in wild emmer, i.e. before domestication. The lack of genetic diversity in *NAM-B1* among accessions carrying the +1 bp allele suggests that the insertion did not happen long before domestication and argues against a soft sweep at *NAM-B1*. The extensive linkage disequilibrium around *NAM-B1* and the reduced genetic diversity strongly suggests a hard selective sweep on the +1 bp allele even as the region does not test significantly for selection. We therefore consider the selection criterion to also be fulfilled for *NAM-B1*.

The final criterion posits that causative alleles should be at or near fixation in all lineages from a single domestication event [18]. In this and previous studies [19] *NAM-B1* has been shown to be fixed for the null alleles in durum. Bread wheat, could be argued to be nearly fixed for the null alleles with the WT allele only persisting in Fennoscandian spring wheat [30]. It can thus be argued that in terms of durum and bread wheat *NAM-B1* is a bona fide domestication gene as has been suggested [17, 35–37]. In contrast, for domesticated emmer the WT allele was present in close to a third of the genotyped accessions, refuting the status of *NAM-B1* as a domestication gene in the evolutionary lineage that gave rise to domesticated emmer. Durum is generally believed to have developed from emmer [4, 10, 12]. Under such a scenario it is not clear whether *NAM-B1* can be called a domestication gene for tetraploid wheats. In comparison, the above-mentioned *tb1* can be classified as a domestication gene since the alleles differ between wild and domesticated maize [57, 58]. In contrast, both wild-type and null-alleles of *Wx* occurs in domesticated rice, suggesting that *Wx* is instead a diversification gene [33]. Our finding of both functional and null alleles in domesticated emmer wheat suggests that *NAM-B1* should be considered a diversification gene for tetraploid wheats. Selection on *NAM-B1* null alleles would have begun already in domesticated emmer, but intensified with the development of durum where the null alleles finally became fixed.

The strongly contrasting fates of the null alleles in bread wheat and durum compared to emmer raise the question of why the WT allele is still present in emmer. Although *NAM-B1* null alleles increase grain weight, the effect on overall yield is not necessarily clear [26]. This may be due to other factors effecting yield that under some conditions compensate for the reduced grain size in WT wheat [61]. Distelfeld, et al. [22] proposed that a delayed senescence would only be beneficial in a mild humid climate where a prolonged grain-filling would be possible and result in larger grain. In a dry environment, prolonged grain-filling would instead be restricted by limited water availability and the yield effects of delayed senescence would be small or negative. Carter, et al. [62] did notice earlier senescence among wheat with the WT allele grown under greenhouse conditions, but not in plants grown in the field in an area with high mid-season growing temperatures.

It is possible that the selection of genes affecting senescence rate and grain size differ depending on climate. That this may be the case for *NAM-B1* is supported by the fact that in bread wheat the WT allele occurs only in Fennoscandian spring wheats, where the benefits of a fast maturation seem to have outweighed the yield effects of the null alleles [30]. The case is much less straightforward when it comes to the domesticated emmers in this study. The effects of genotype - environment interactions on seed size and yield have not yet been tested in emmer wheat. However, we note that accessions carrying the WT allele originated from Spain in the west to Iran in the east and from Ethiopia in the south to Belarus in the north, while accessions with null alleles originated from a similar area (Additional file 1). Environmental selection pressures acting similarly over this vast area to preserve the WT allele seem unlikely.

An alternative explanation could be gene flow between wild and domesticated emmer. Many of the emmers carrying the WT allele originated from areas where wild emmers grow (Iran: *N* = 5, Turkey: *N* = 3, Additional file 1) [5]. Gene flow is supported by the clustering of WT emmers among the wild emmers in the *NAM-B1* gene tree (Fig. 3b). Gene flow between wild emmer and domesticated emmer from central Europe or Africa, however, seems less likely. Durum wheat has been shown to have limited geographic structure in the Mediterranean area [12], and a dynamic history of wheat farming has been suggested [48]. If this is the case for emmer, long distance seed trade could have spread WT emmer far from areas where gene flow from wild emmer can occur. Limited selection for seed size may have allowed genetic drift to preserve the WT allele in some populations.

## Conclusion

In this study we have shed light on the evolutionary history of *NAM-B1*. We have shown that the deletion allele covers several 100 kb and that the +1 bp likely arose among wild emmer. The +1 bp allele was then the target of a selective sweep, also extending over several 100 kb, and possibly stronger than the selection acting on the *tb1* locus in maize. Both null alleles occur in domesticated emmer and durum and in the latter the null allele seems to be fixed. The frequent occurrences of the WT allele among domesticated emmer, however, raises questions as to whether the *NAM-B1* gene is a bona fide tetraploid wheat domestication gene. Instead we propose it to be a diversification gene in this species.

## Additional files

Additional file 1: List of wheat accessions and their sites of origin. (XLSX 14 kb)

Additional file 2: List of PCR amplified fragments. Primer sequences and PCR conditions. (XLSX 10 kb)

Additional file 3: Neighbor-joining trees of wheat sequences in fragments surrounding *NAM-B1* and reference genes. (A) Reference gene 11B. (B) Reference gene Gsp1B. (C) Reference gene MdhA. (D) Reference gene Mp7A. (E) Fragment t_10kb. (F) Fragment c_7kb. (G) Fragment c_174kb. (H) Fragment c_Yr36 + 3. (I) Fragment c_Yr36 + 52. (PDF 410 kb)

Additional file 4: Nucleotide diversity and Theta W Tables. A) Nucleotide diversity (π). B) Theta W. (XLSX 11 kb)

Additional file 5: Neutrality tests. Tables over the resulting test values from the neutrality tests. (A) Tajima’s D. (B) Fu and Li’s D and D^*^. (C) Fu and Li’s F and F^*^. (XLSX 14 kb)

## Abbreviations

+1 bp: one-basepair insertion
cM: centiMorgan
del: Deletion
kb: Kilobasepair
PCR: Polymerase Chain Reaction
SNP: Single Nucleotide Polymorphism
*tb1*: *teosinte brached 1*
WT: Wild-type
*Wx*: *Waxy*
