# Supplementary material for: Evolutionary history of the NAM-B1 gene in wild and domesticated tetraploid wheat
Source: BMC Genet. 2017 Dec 20;18:118. doi: 10.1186/s12863-017-0566-7 (PMC5738170; doi:10.1186/s12863-017-0566-7)
Supplement: Supplementary file 3 — Neighbor-joining trees of wheat sequences in fragments surrounding NAM-B1 and reference genes. (A) Reference gene 11B. (B) Reference gene Gsp1B. (C) Reference gene MdhA. (D) Reference gene Mp7A. (E) Fragment t_10kb. (F) Fragment c_7kb. (G) Fragment c_174kb. (H) Fragment c_Yr36 + 3. (I) Fragment c_Yr36 + 52. (PDF 410 kb) [file 12863_2017_566_MOESM3_ESM.pdf]

A 11B

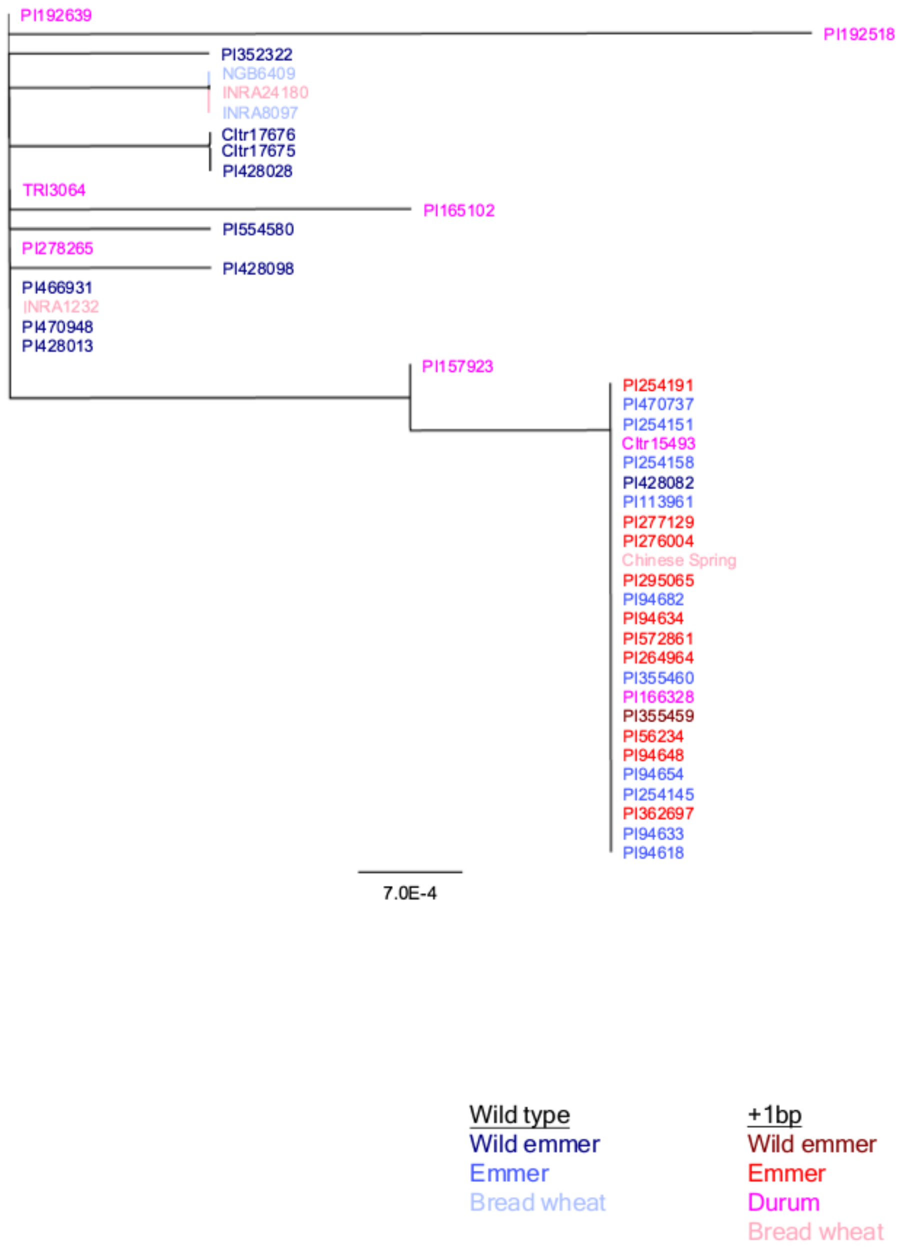
PI192639
PI192518
PI352322
NGB6409
INRA24180
INRA8097
CItr17676
CItr17675
PI428028
TRI3064
PI165102
PI554580
PI278265
PI428098
PI466931
INRA1232
PI470948
PI428013
PI157923
PI254191
PI470737
PI254151
CItr15493
PI254158
PI428082
PI113961
PI277129
PI276004
Chinese Spring
PI295065
PI94682
PI94634
PI572861
PI264964
PI355460
PI166328
PI355459
PI56234
PI94648
PI94654
PI254145
PI362697
PI94633
PI94618
7.0E-4
Wild type
Wild emmer
Emmer
Bread wheat
+1bp
Wild emmer
Emmer
Durum
Bread wheat

B GspB1

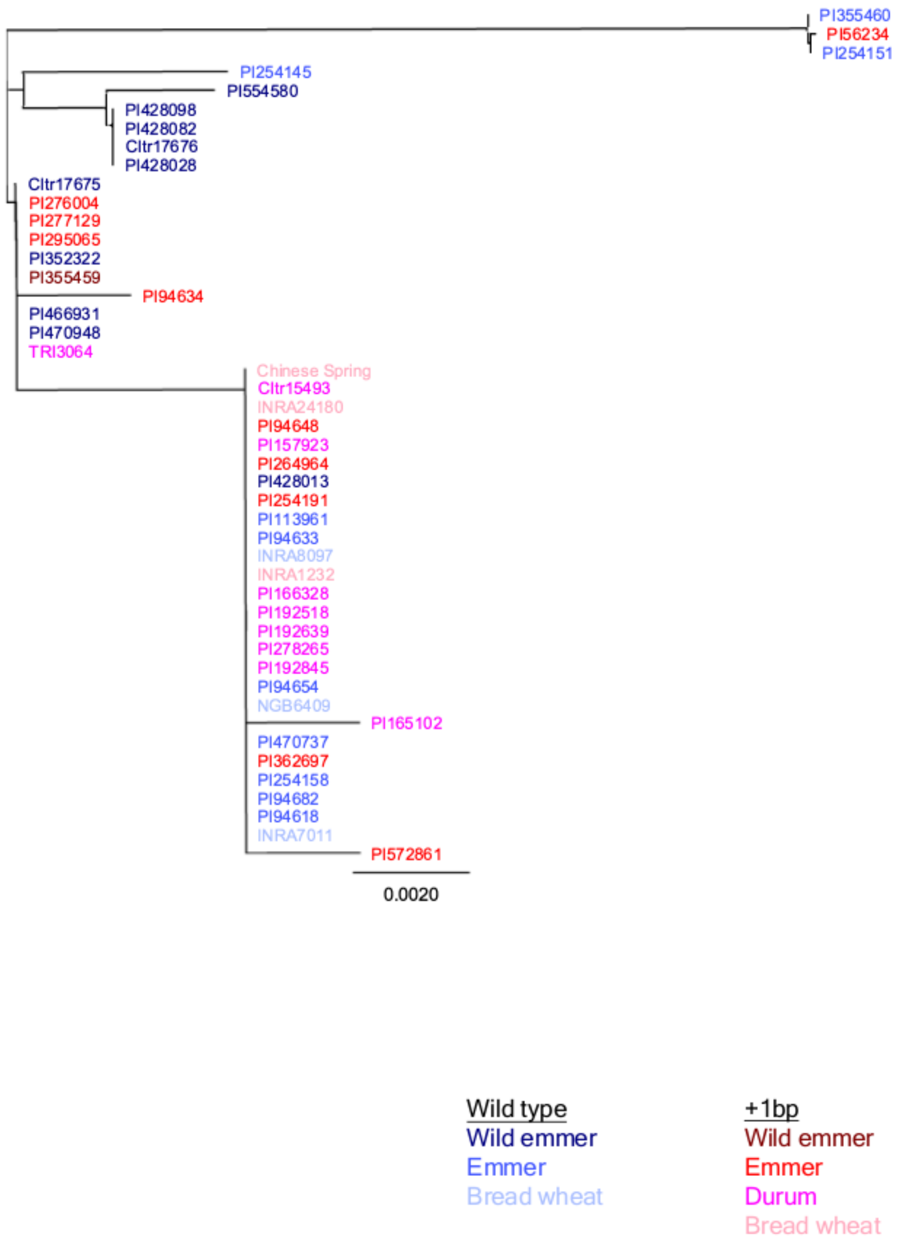
PI355460
PI56234
PI254151
PI254145
PI554580
PI428098
PI428082
Cltr17676
PI428028
Cltr17675
PI276004
PI277129
PI295065
PI352322
PI355459
PI94634
PI466931
PI470948
TRI3064
Chinese Spring
Cltr15493
INRA24180
PI94648
PI157923
PI264964
PI428013
PI254191
PI113961
PI94633
INRA8097
INRA1232
PI166328
PI192518
PI192639
PI278265
PI192845
PI94654
NGB6409
PI165102
PI470737
PI362697
PI254158
PI94682
PI94618
INRA7011
PI572861
0.0020
Wild type
Wild emmer
Emmer
Bread wheat
+1bp
Wild emmer
Emmer
Durum
Bread wheat

C MdhA

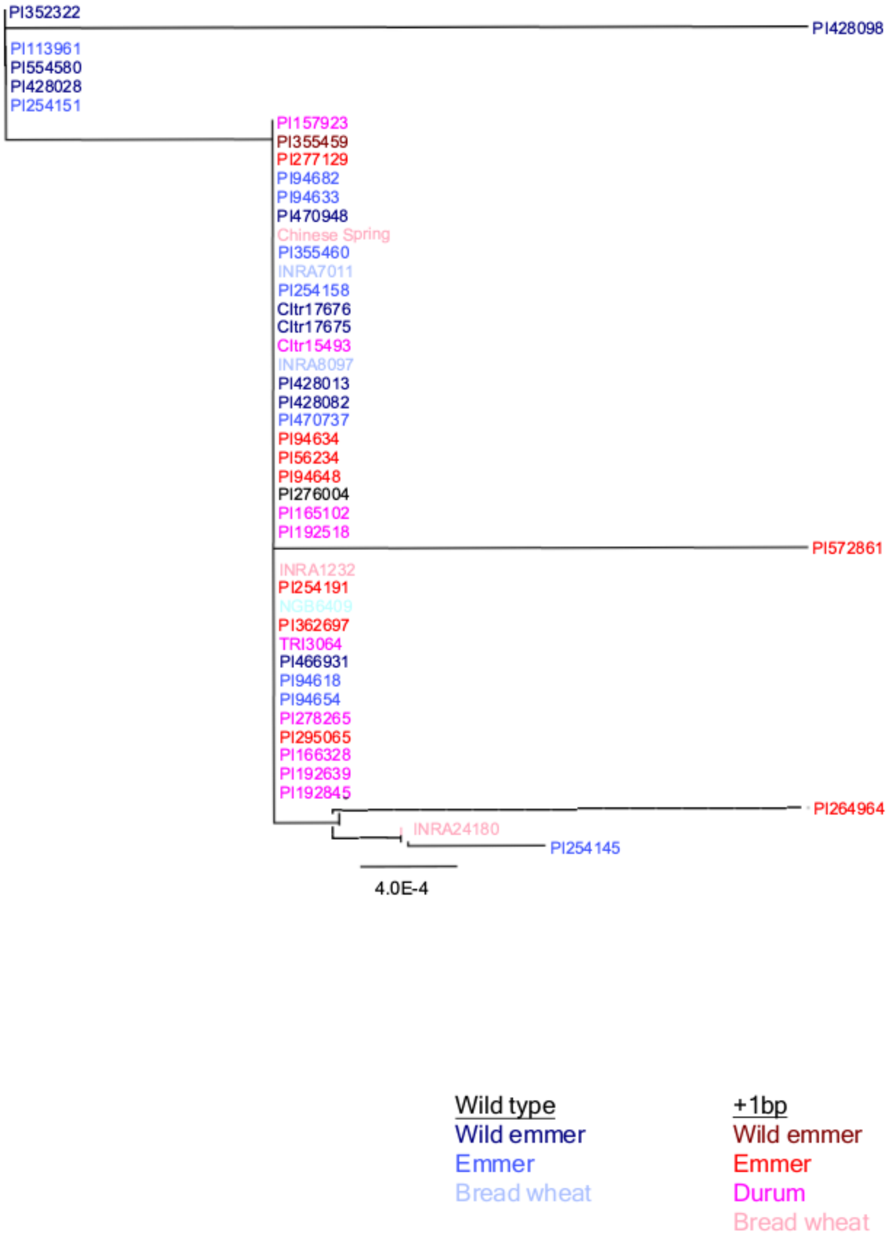
PI352322
PI428098
PI113961
PI554580
PI428028
PI254151
PI157923
PI355459
PI277129
PI94682
PI94633
PI470948
Chinese Spring
PI355460
INRA7011
PI254158
Cltr17676
Cltr17675
Cltr15493
INRA8097
PI428013
PI428082
PI470737
PI94634
PI56234
PI94648
PI276004
PI165102
PI192518
PI572861
INRA1232
PI254191
NGB6409
PI362697
TRI3064
PI466931
PI94618
PI94654
PI278265
PI295065
PI166328
PI192639
PI192845
PI264964
INRA24180
PI254145
4.0E-4
Wild type
Wild emmer
Emmer
Bread wheat
+1bp
Wild emmer
Emmer
Durum
Bread wheat

D Mp7A

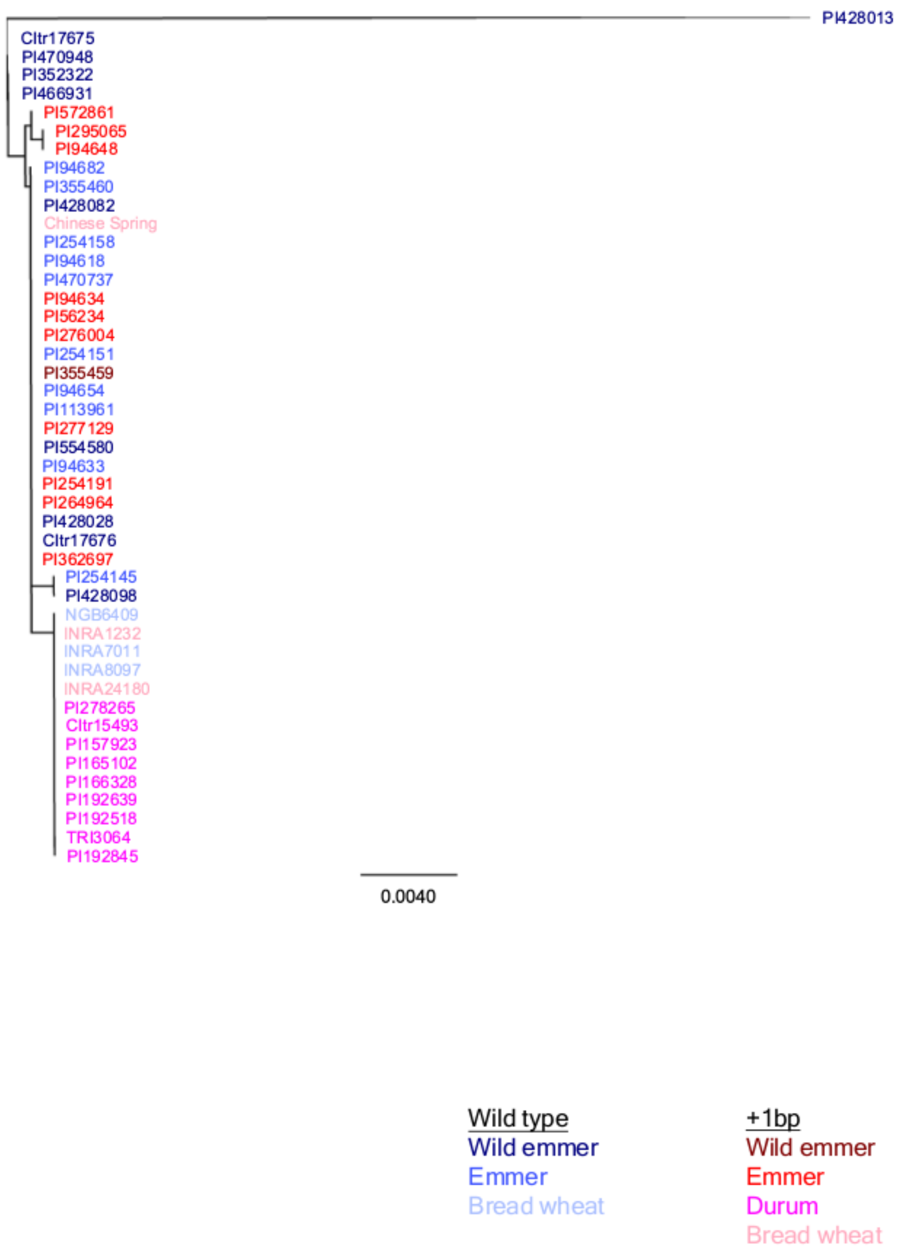
PI428013
Cltr17675
PI470948
PI352322
PI466931
PI572861
PI295065
PI94648
PI94682
PI355460
PI428082
Chinese Spring
PI254158
PI94618
PI470737
PI94634
PI56234
PI276004
PI254151
PI355459
PI94654
PI113961
PI277129
PI554580
PI94633
PI254191
PI264964
PI428028
Cltr17676
PI362697
PI254145
PI428098
NGB6409
INRA1232
INRA7011
INRA8097
INRA24180
PI278265
Cltr15493
PI157923
PI165102
PI166328
PI192639
PI192518
TRI3064
PI192845
0.0040
Wild type
Wild emmer
Emmer
Bread wheat
+1bp
Wild emmer
Emmer
Durum
Bread wheat

E t_10kb

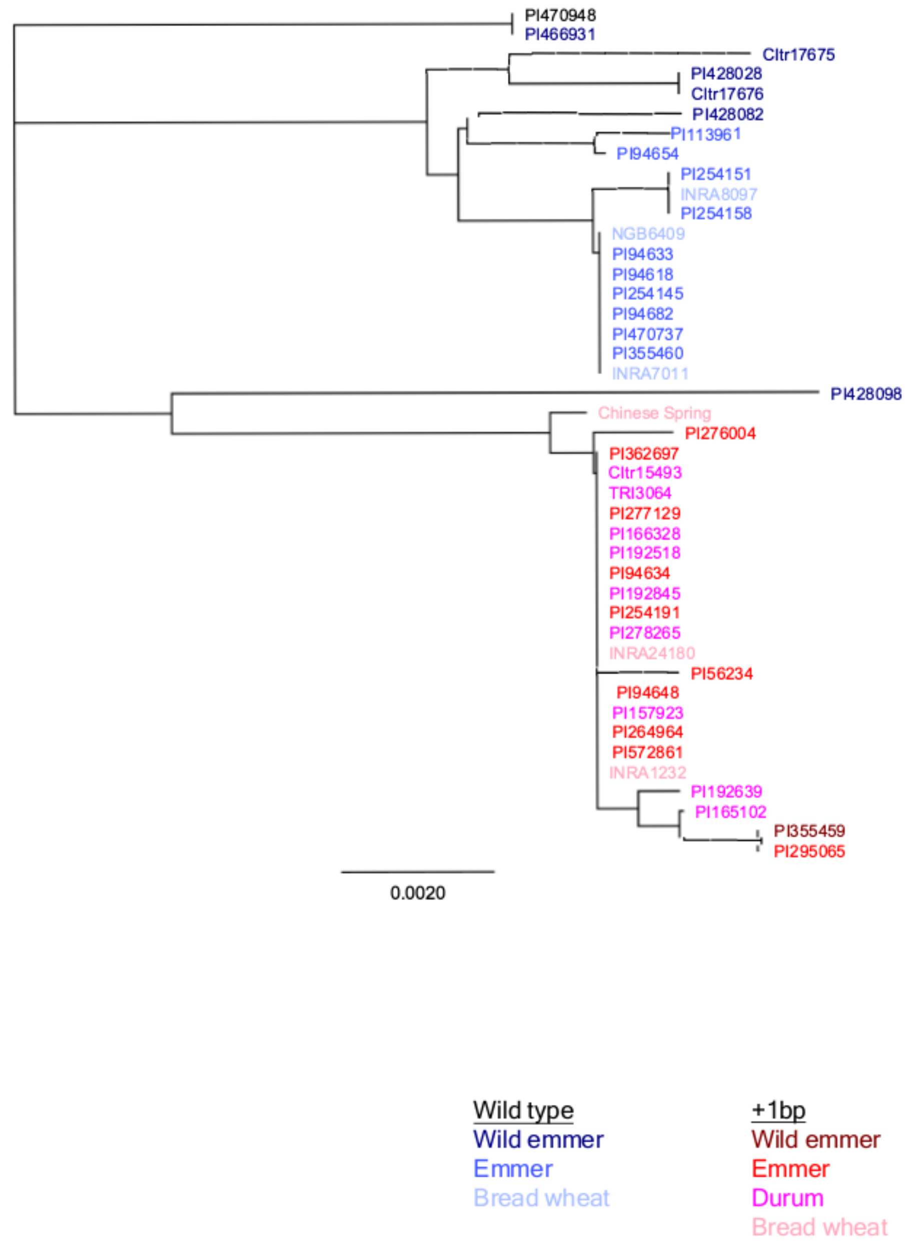
PI470948
PI466931
Cltr17675
PI428028
Cltr17676
PI428082
PI113961
PI94654
PI254151
INRA8097
PI254158
NGB6409
PI94633
PI94618
PI254145
PI94682
PI470737
PI355460
INRA7011
PI428098
Chinese Spring
PI276004
PI362697
Cltr15493
TRI3064
PI277129
PI166328
PI192518
PI94634
PI192845
PI254191
PI278265
INRA24180
PI56234
PI94648
PI157923
PI264964
PI572861
INRA1232
PI192639
PI165102
PI355459
PI295065
0.0020
Wild type
Wild emmer
Emmer
Bread wheat
+1bp
Wild emmer
Emmer
Durum
Bread wheat

F

c_7kb

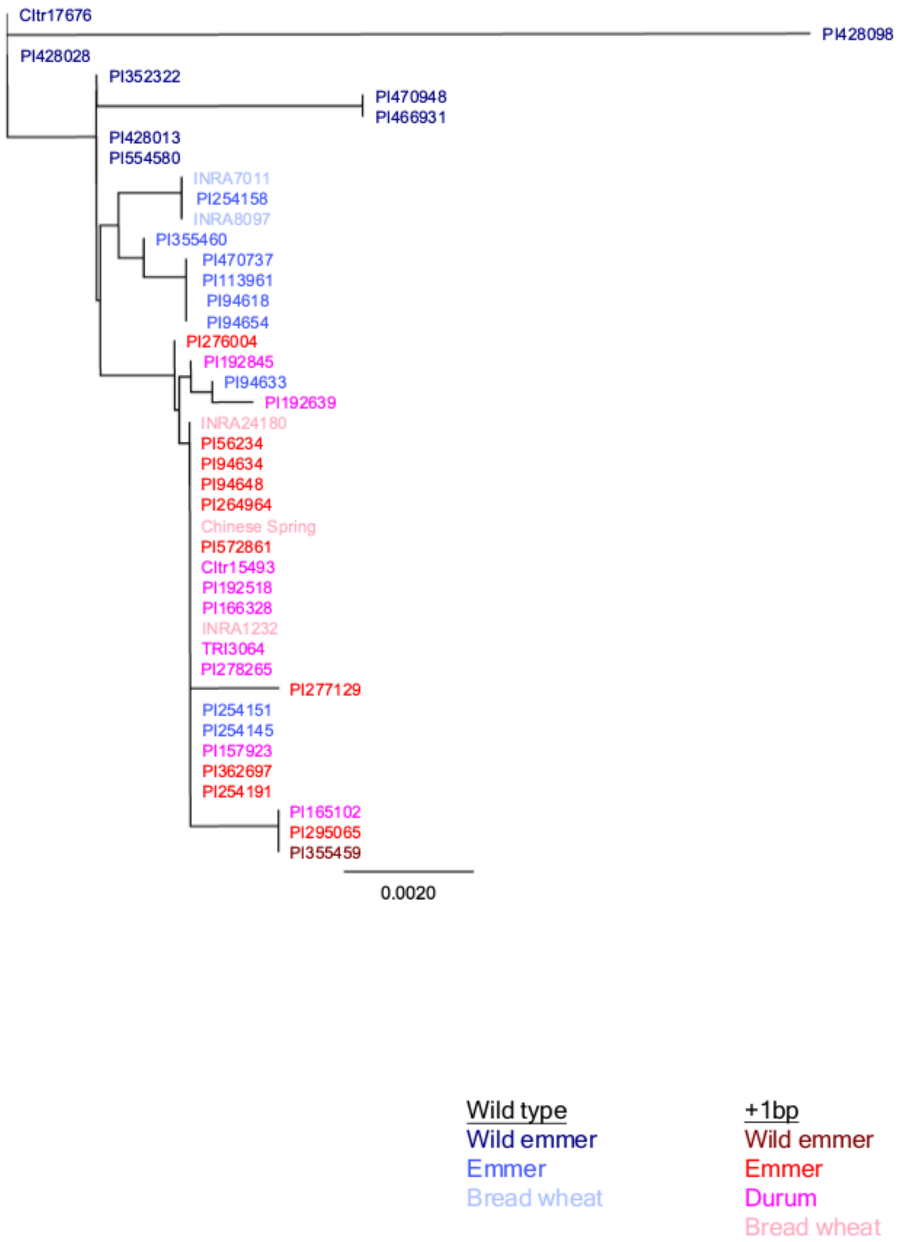
Cltr17676
PI428098
PI428028
PI352322
PI470948
PI466931
PI428013
PI554580
INRA7011
PI254158
INRA8097
PI355460
PI470737
PI113961
PI94618
PI94654
PI276004
PI192845
PI94633
PI192639
INRA24180
PI56234
PI94634
PI94648
PI264964
Chinese Spring
PI572861
Cltr15493
PI192518
PI166328
INRA1232
TRI3064
PI278265
PI277129
PI254151
PI254145
PI157923
PI362697
PI254191
PI165102
PI295065
PI355459
0.0020
Wild type
Wild emmer
Emmer
Bread wheat
+1bp
Wild emmer
Emmer
Durum
Bread wheat

G c_174kb

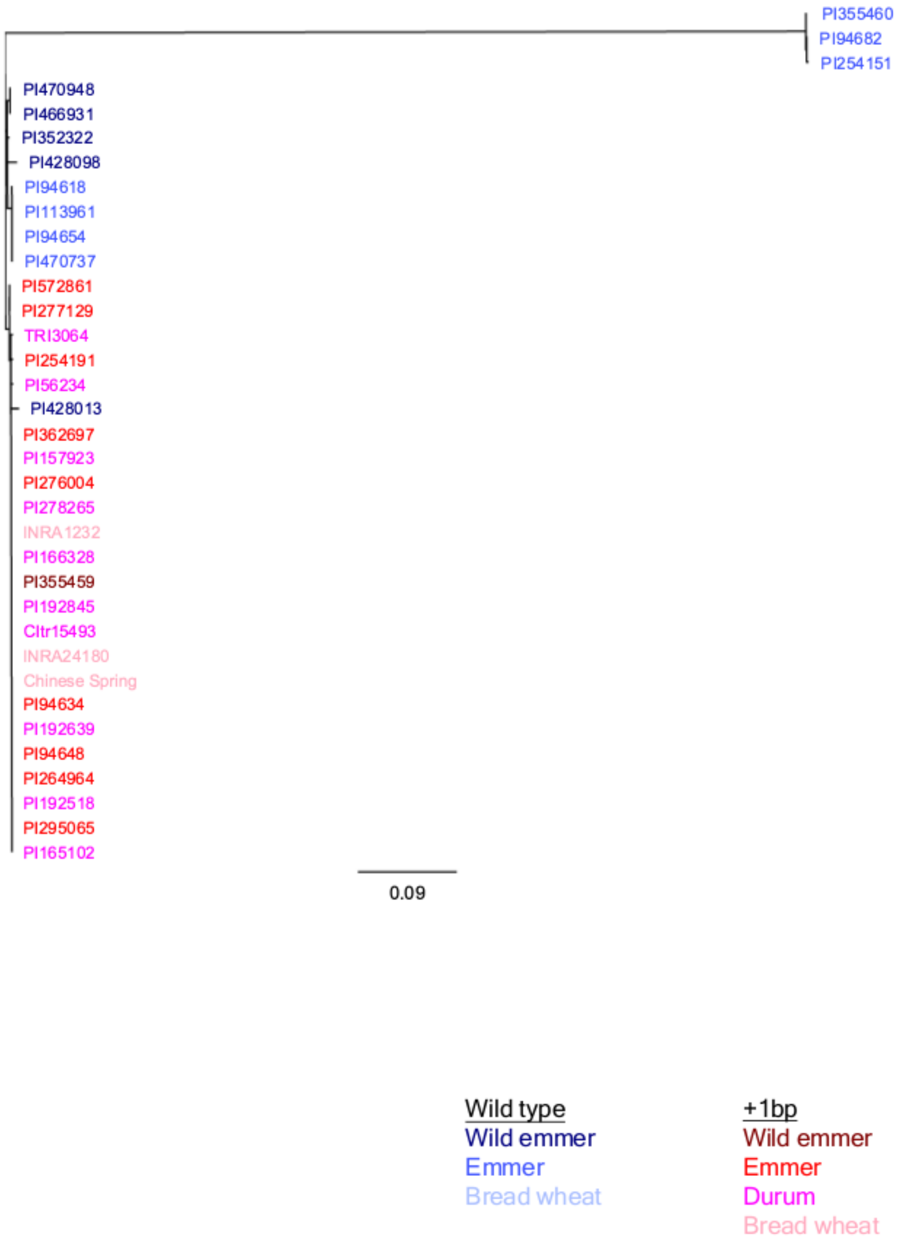
PI355460
PI94682
PI254151
PI470948
PI466931
PI352322
PI428098
PI94618
PI113961
PI94654
PI470737
PI572861
PI277129
TRI3064
PI254191
PI56234
PI428013
PI362697
PI157923
PI276004
PI278265
INRA1232
PI166328
PI355459
PI192845
CItr15493
INRA24180
Chinese Spring
PI94634
PI192639
PI94648
PI264964
PI192518
PI295065
PI165102
0.09
Wild type
Wild emmer
Emmer
Bread wheat
+1bp
Wild emmer
Emmer
Durum
Bread wheat

H c_Yr36+3

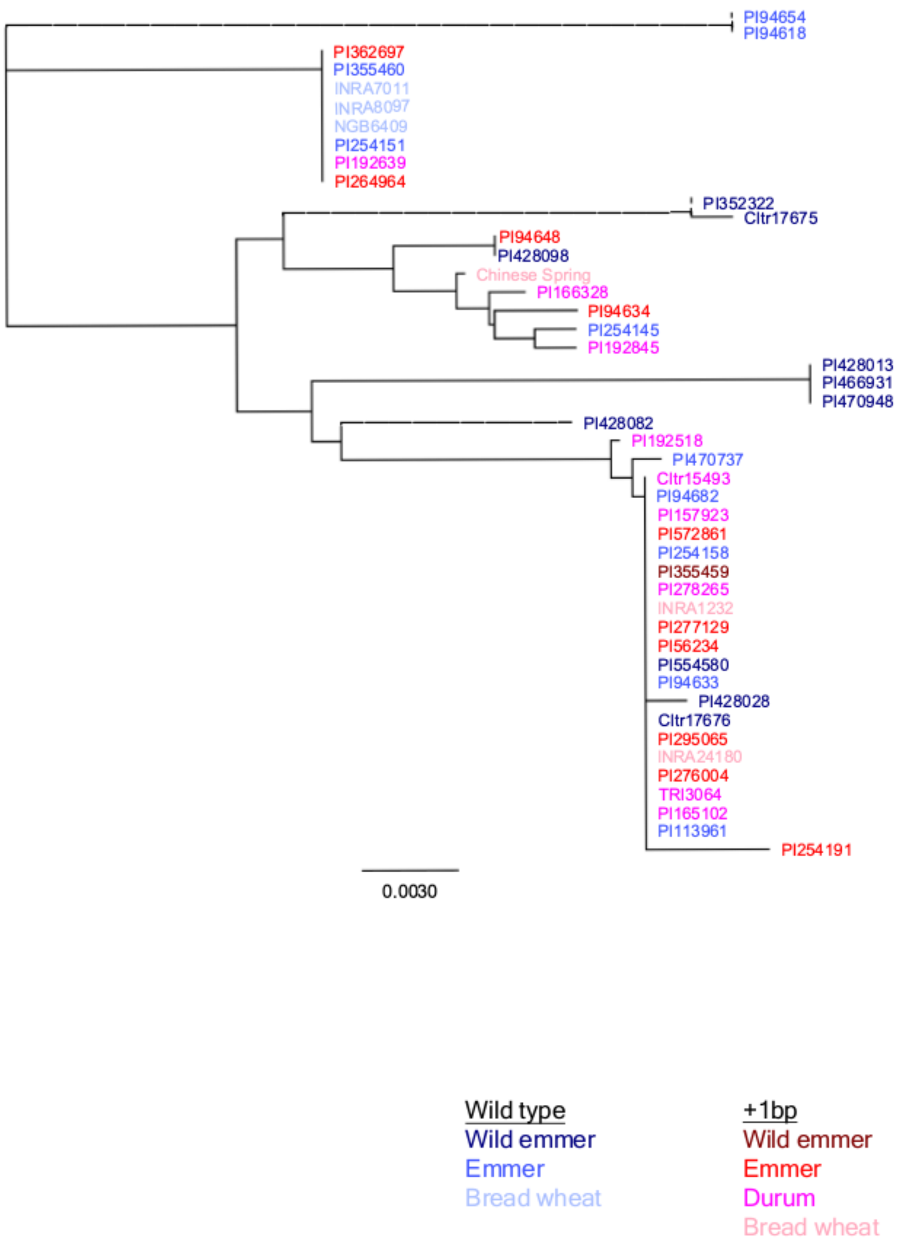
PI94654
PI94618
PI362697
PI355460
INRA7011
INRA8097
NGB6409
PI254151
PI192639
PI264964
PI352322
Cltr17675
PI94648
PI428098
Chinese Spring
PI166328
PI94634
PI254145
PI192845
PI428013
PI466931
PI470948
PI428082
PI192518
PI470737
Cltr15493
PI94682
PI157923
PI572861
PI254158
PI355459
PI278265
INRA1232
PI277129
PI56234
PI554580
PI94633
PI428028
Cltr17676
PI295065
INRA24180
PI276004
TRI3064
PI165102
PI113961
PI254191
0.0030
Wild type
Wild emmer
Emmer
Bread wheat
+1bp
Wild emmer
Emmer
Durum
Bread wheat

I c_Yr36+52

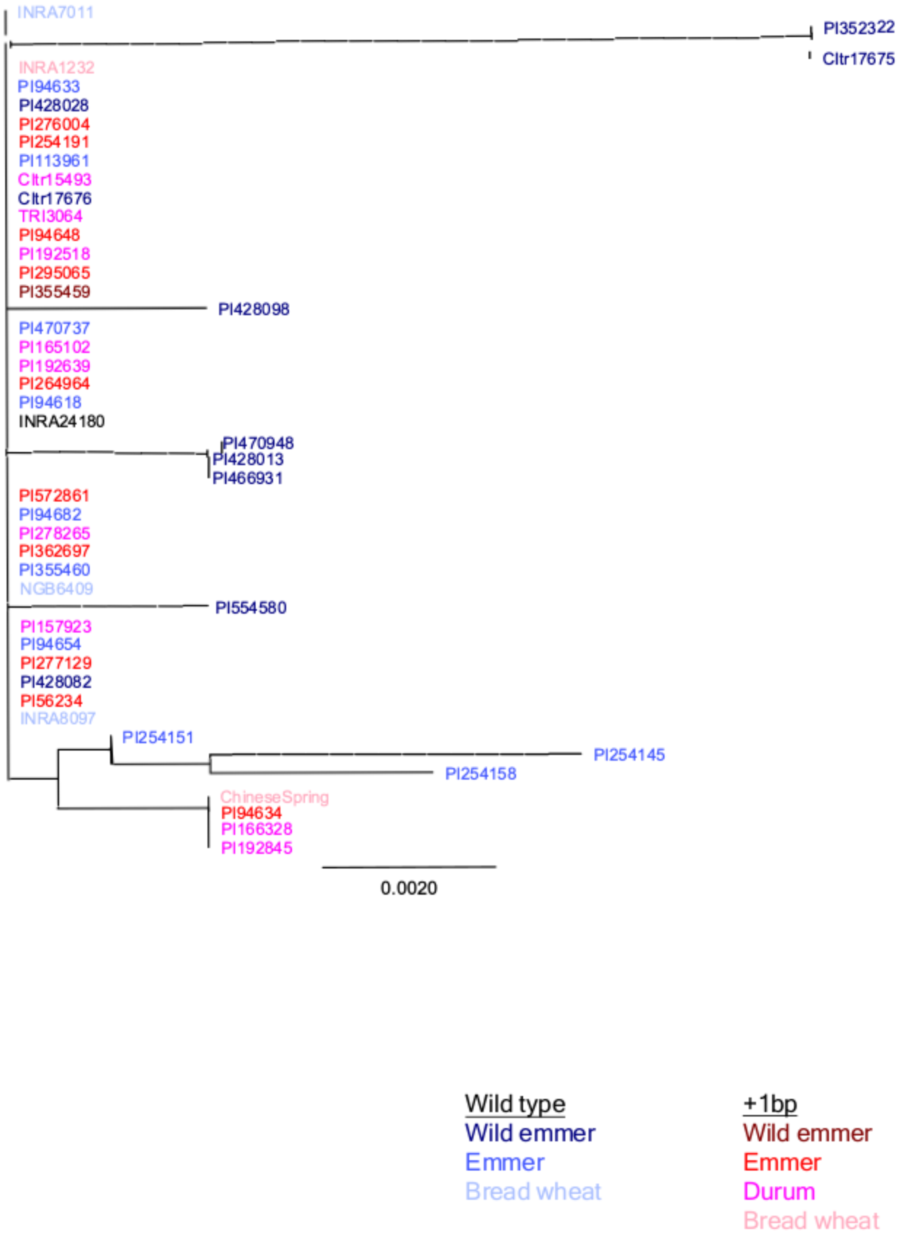
INRA7011
PI352322
Cltr17675
INRA1232
PI94633
PI428028
PI276004
PI254191
PI113961
Cltr15493
Cltr17676
TRI3064
PI94648
PI192518
PI295065
PI355459
PI428098
PI470737
PI165102
PI192639
PI264964
PI94618
INRA24180
PI470948
PI428013
PI466931
PI572861
PI94682
PI278265
PI362697
PI355460
NGB6409
PI554580
PI157923
PI94654
PI277129
PI428082
PI56234
INRA8097
PI254151
PI254145
PI254158
ChineseSpring
PI94634
PI166328
PI192845
0.0020
Wild type
Wild emmer
Emmer
Bread wheat
+1bp
Wild emmer
Emmer
Durum
Bread wheat
